# Sustainable Nutrient Recovery from Wastewater Mixture to Optimize Microalgal Lipid Production: A Vision of Zero Water Footprint

**DOI:** 10.3390/bioengineering12121291

**Published:** 2025-11-24

**Authors:** Marco Alberto Mamani Condori, Danae Colque Ollachica, Abel Roberto Ccapa Loncone, José C. M. Pires, Ana María Gagneten

**Affiliations:** 1 Facultad de Ingeniería de Procesos, Universidad Nacional de San Agustín de Arequipa, Arequipa 04000, Peru; 2 LEPABE, ALiCE, Faculty of Engineering, University of Porto, Rua Dr. Roberto Frias, 4200-465 Porto, Portugal; 3 Laboratorio de Ecotoxicología, Facultad de Humanidades y Ciencias, Universidad Nacional del Litoral, RN 168, Km 0, Santa Fe S3000ADQ, Argentina

**Keywords:** sustainable bioprocess, mixotrophic cultivation, wastewater blending, nutrient removal, circular economy

## Abstract

In this study, two native microalgae, *Chlorella* sp. MC18 (CH) and *Scenedesmus* sp. MJ23-R (SC) were cultivated in bubble column photobioreactors for wastewater treatment. Domestic wastewater (DWW) was used as the main culture medium, alone (100%) and blended (10%) with vinasse, whey, or agro-food waste (AFW), respectively. Both species thrived in 100% DWW, achieving significantly high removal efficiencies for chemical oxygen demand, total nitrogen, and total phosphorus. Mineral removal exceeded 90% in all blended systems, highlighting the strong nutrient uptake capacity of both strains. The maximum specific growth rate (*µ_max_*) in 100% DWW was higher for SC than in standard BG11 medium, and supplementation with vinasse, whey, or AFW further increased *µ_max_* for both species. Blending DWW significantly enhanced microalgal biomass and lipid production compared to 100% DWW. Lipid production (max., 374 mg L^−1^), proximate lipid composition (max., 30.4%), and lipid productivity (max., 52.9 mg L^−1^ d^−1^) significantly increased in all supplemented cultures relative to DWW alone, demonstrating the potential of co-substrate supplementation to optimize microalgal cultivation. This study contributes to reducing the water footprint and fills a gap in the bioprocessing potential of algae-based systems, highlighting wastewater blending as a circular economy-aligned approach that supports sustainable bioprocesses and resource recovery.

## 1. Introduction

Human and industrial activities generate a huge amount of sludge globally, characterized by high organic content, although some progress has been made to reduce the environmental footprint. Jones et al. [1] estimated that 48% of global wastewater production is released to the environment untreated, which is substantially lower than previous estimates of approximately 80%. Moreover, approximately 40.7 × 10^9^ m^3^ yr^−1^ of treated wastewater is intentionally reused [2]. Domestic wastewater (DWW), rich in nutrients, represents a valuable yet underutilized resource for agriculture. Globally, more than 45 million tons are generated annually, with 70% discharged into the environment untreated or insufficiently treated [2,3], raising critical concerns about sustainable disposal and reuse, particularly in developing countries where infrastructure and resources remain limited, thereby posing a risk to ecosystems. Given that DWW, distillery wastewater (vinasse), dairy wastewater (whey), and agro-food waste (AFW) are increasingly generated and projected to continue growing in the coming years [4,5,6,7], their management demands urgent attention.

The growing need for wastewater recovery and the mitigation of eutrophication underscore the urgency of accelerating nutrient recovery, which is considered one of the greatest sustainability challenges of the twenty-first century [8]. Addressing this issue requires a paradigm shift from wastewater treatment plants (WWTPs) to water resource recovery facilities (WRRFs), supported by a thorough understanding of available recovery and recycling technologies [9]. Within this framework, numerous efforts have been directed toward enhancing microalgal biomass and lipid production using specific wastewaters, thereby achieving the dual goals of wastewater treatment and resource recovery through microalgal systems. Other authors already view food-sector wastewater as a cultivation medium for lipid-rich algae [10]. However, some issues remain unsolved, especially regarding DWW, vinasse, whey, and AFW. To date, there is a lack of comparative studies on the synergistic effects of these residual mixtures on both nutrient removal efficiency and lipid productivity for *Chlorella* sp. and *Scenedesmus* sp. species. A key limitation is that nutrient uptake mechanisms vary with wastewater composition [11], directly influencing both biomass accumulation and lipid productivity. Moreover, most approaches require wastewater dilution or complex pretreatment to enable algal growth [12,13,14]. Traditional WWTPs use mechanical methods [15], while newer approaches—such as electrocoagulation and constructed wetlands—aim to adapt undiluted wastewaters for algal growth. Although freshwater dilution is common, it is unsustainable. On the other hand, some authors claim that pretreatment is vital for algal growth and pollutant removal [16,17]. Unlike the conventional methods described, this study employs simple physical steps (i.e., sedimentation, centrifugation, and filtration), such as efficient and non-polluting pretreatment steps, combined with wastewater blending, to minimize freshwater consumption and enhance nutrient recovery from DWW and agro-industrial effluents.

Derco et al. [18] and Satiro et al. [19] both support the use of blended wastewater sources for improved treatment outcomes. Derco et al. [18] highlighted how combining different wastewaters enhances nutrient recovery and helps meet strict effluent standards, while in the comprehensive review by Satiro et al. [19], the authors provide a strong theoretical framework explaining how microalgae–bacteria consortia enhance organic matter degradation, oxygen–carbon cycling, and lipid accumulation, while improving biomass settling and granulation. As discussed in this article, these consortia enable a natural exchange of oxygen and carbon dioxide between partners, supporting nitrogen and phosphorus assimilation and the formation of lipid-rich biomass. This symbiotic metabolism minimizes external aeration requirements and supports circular bioprocesses aligned with low-energy and zero-emission wastewater treatment, from a zero-water footprint perspective [8,20]. Recently, Sátiro et al. [21] demonstrated nutrient recovery and biomass/lipid optimization in wastewater-based algal cultivation, providing experimental evidence that activated-sludge inoculation enhances microalgae–bacteria symbiosis within pilot-scale high-rate ponds. The improved nutrient removal efficiency and biomass settling obtained in this work are consistent with previous known biochemical and microbial interactions [19], highlighting that our results are not only empirical but mechanistically explained by algal–bacterial symbiosis.

Another challenge associated with this approach is that wastewater blending must achieve a proper nutrient balance to optimize algal biomass production. This aspect is also addressed in the present study, by comparing the performance of *Chlorella* sp. and *Scenedesmus* sp., while simultaneously optimizing lipid production. In the present study, we combined DWW with vinasse, whey, and AFW to optimize nutrient balance and lipid productivity. A similar objective was pursued by Tang et al. [22], who blended two types of wastewaters (municipal wastewater and anaerobic membrane bioreactor effluent) in varying proportions. They monitored microalgal biomass growth, pollutant removal, and lipid production, concluding that adjusting the blend composition can enhance both nutrient removal and lipid accumulation, rather than solely improving treatment efficiency.

Most studies on microalgal lipid production have focused on synthetic culture media or single-source effluents [14]. While some recent efforts have examined blended effluents [23,24], the issue remains far from resolved and constitutes a significant knowledge gap. To date, no research has evaluated the blending of DWW with vinasse, whey, and AFW for biomass and lipid production, using two key-freshwater microalgal species cultivated in bubble column photobioreactors (BC-PBRs). This approach reduces the production costs of biomass and high-value bioproducts, while enhancing the economic viability of algal bioprocesses by utilizing wastewater, an easily available and low-cost nutrient source. Furthermore, the study offers practical insights into the integration of algal bioprocesses for waste treatment as a scalable and sustainable strategy, reinforcing the principles of the circular economy [5,25,26]. The aim of this study is to evaluate the potential of *Chlorella* sp. MC18 and *Scenedesmus* sp. MJ23-R in BC-PBRs for sustainable nutrient recovery (mainly nitrogen and phosphorus) from DWW, vinasse, whey and AFW blended wastewaters, and to optimize microalgal biomass yield and lipid production, with the overarching goal of achieving a zero-water-footprint process. We hypostatize that *Chlorella* and *Scenedesmus* cultivated in batch-operated BC-PBRs using blended wastewaters will demonstrate enhanced microalgal growth kinetics, efficient nutrient recovery, and significant contaminant elimination, leading to optimized lipid yield and biomass valorization. Furthermore, scaling BC-PBRs under controlled conditions will improve system efficiency and support the sustainable integration of microalgae-based bioprocesses for wastewater treatment and bioresource production.

## 2. Materials and Methods

### 2.1. Microalgae Species and Cultivation

Freshwater microalgae, *Chlorella* sp. (MC18) and *Scenedesmus* sp. (MJ23-R), were obtained from a private culture collection of the Biochemistry and Molecular Biology Laboratory at the National University of Saint Augustine (Arequipa, Peru) [8]. Pure cultures of each microalga were initially grown in Erlenmeyer flasks containing 1 L of sterile BG11 medium [27], autoclaved for 15 min at 121 °C. After 5 days of incubation, when cell density reached ~2.0 × 10^7^ cells mL^−1^, inoculant was collected by centrifugation (15,025 RCF-max, 10 min, 4 °C; Rotanta 460R, Hettich Zentrifugen, Tuttlingen, Germany). Biomass pellets were washed three times with sterile distilled water and centrifuged again. Exponentially growing microalgal cells were then inoculated into different DWW formulations for pre-acclimation in 1 L bubble column photobioreactors (BC-PBRs), until reaching an initial concentration of 1.0 × 10^6^ cells mL^−1^ or 10 mg_DW_ L^−1^. The acclimation process for both microalgae was conducted over at least two months (equivalent to five cycles) to ensure cellular homeostasis and achieve ionic balance in *Chlorella* sp. and *Scenedesmus* sp. [28]. Shortly, each cultivation cycle involved a 10-day incubation period, after which the newly generated inocula were used for subsequent treatment cultures. The specific criteria for the serial acclimation process were established based on findings from previous studies [8,28,29]; that is, *Chlorella* or *Scenedesmus* were considered to have reached cellular stability when growth kinetics plateaued. Notably, five cultivation cycles were sufficient to ensure the acclimation of the microalgae to the culture system. This strategy was essential for maximizing nutrient recovery and valuable biomass production. At the logarithmic phase of the final cycle, each culture broth was harvested and used as inoculum to assess the individual tolerance of the microalgae to the DWW formulations (see Section 2.4).

Flasks and BC-PBRs were aerated with a continuous airflow at 0.25 and 0.5 vvm (air volume per liquid volume per minute), respectively, while maintaining a stable temperature of 24 ± 2 °C. Illumination was provided by cool daylight LED tubes (Ecofit E Mains, Philips Lighting, Eindhoven, The Netherlands) with a photosynthetically active radiation (PAR) of 60 ± 6 μmol photons m^−2^ s^−1^ at the surface of the flasks and BC-PBRs, under a 12:12 h light/dark (L/D) cycle. Additionally, all chemicals used in this work were of analytical grade, purchased from commercial sources, and applied without further purification.

### 2.2. Characterization of Different Wastewaters

In this study, domestic wastewater (DWW), distillery wastewater (vinasse), raw dairy wastewater (whey), and agro-food waste (AFW) were used. DWW samples were collected from a local community (Espinar Province, Cusco, Peru) with a flow rate of 3.14 ± 0.15 m^3^ h^−1^. Suspended particulate matter was removed by sedimentation. Tang et al. [22] filtered municipal wastewater through filter paper (with a pore size of 1–3 µm) to avoid the negative impacts of particles in the wastewater on microalgae growth. Sugarcane vinasse was obtained directly from a distillation tower at 90 °C in a sugar and ethanol agroindustry and cooled to ambient temperature (~15 °C). Raw whey was collected after the cheese coagulation process at 35 °C in an artisanal dairy facility. AFW extracts were prepared from spoiled whole fruits, following the detailed methodology described previously [5].

As an efficient, cost-effective, and free of additional chemical agents pretreatment for clarification, vinasse, whey, and AFW samples were centrifuged (15,025 RCF-max, 10 min, 4 °C) to preserve the integrity of essential macro- and micronutrients, crucial for the growth of *Chlorella* sp. MC18 and *Scenedesmus* sp. MJ23-R. Finally, to inhibit heterotrophic proliferation and maintain the chemical composition of the medium, wastewater samples were stored at −20 °C until use. The characteristics of DWW, vinasse, whey, and AFW supernatants are presented in Table 1.

### 2.3. Design of the Bubble Column Photobioreactor

**Figure 1:**
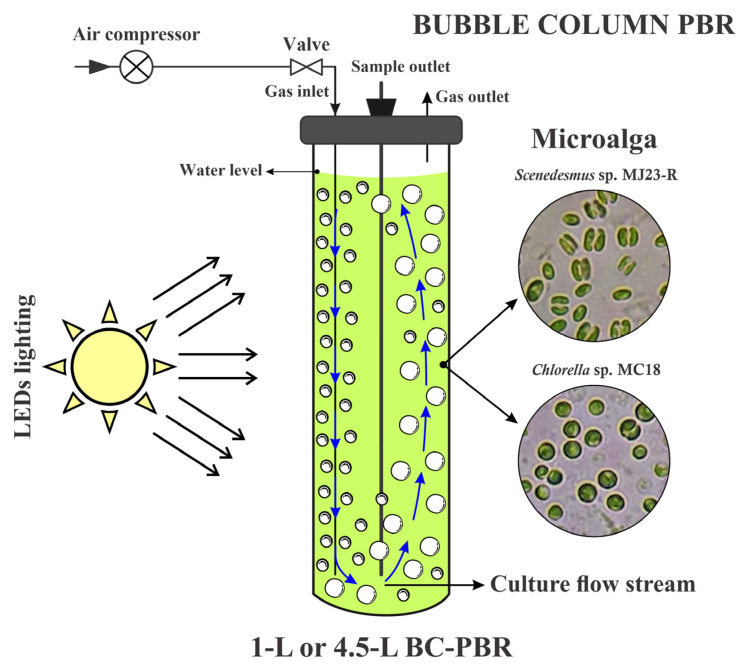
Schematic design of the batch culture system for *Chlorella* sp. MC18 and *Scenedesmus* sp. MJ23-R in bubble column photobioreactors (BC-PBRs).

Based on hydrodynamic principles and the stability of operational parameters, two bubble column photobioreactors (BC-PBRs) of different scales were designed: a 1 L BC-PBR and a 4.5 L BC-PBR (see Figure 1). The set of transparent glass BC-PBRs, with a thickness of 2.5–3 mm, was used for batch cultivation of *Chlorella* sp. MC18 or *Scenedesmus* sp. MJ23-R. These photobioreactors consisted of vertical cylindrical structures with internal diameters of 7.3 cm and 11.2 cm, total heights of 24.5 cm and 46.1 cm, and culture medium heights of 19.2 cm and 40.7 cm, providing working volumes of 0.8 L and 4 L, respectively. Illumination was applied from above, resulting in a surface illumination area of 44.03 × 10^−3^ m^2^ for the 1 L BC-PBR and 143.21 × 10^−3^ m^2^ for the 4.5 L BC-PBR.

A matrix of 16 W cool daylight LED tubes (Ecofit E Mains, Philips Lighting, Eindhoven, The Netherlands), providing PAR irradiance in the range of 60–120 μmol photons m^−2^ s^−1^ under a 12:12 h light/dark (L/D) cycle, supplied illumination to the surfaces of both photobioreactors. The cultures were aerated from the bottom of the BC-PBRs at a constant airflow of 0.5 vvm, delivered by a compressor injecting atmospheric CO_2_ (0.04%) through a 10.0 ± 0.2 mm diffuser, ensuring efficient generation and dispersion of fine gas bubbles. Each BC-PBR was sealed with a hermetic polyvinyl chloride cover, equipped with three polypropylene tubing lines for sample withdrawal, an air inlet to the culture, and an air purge.

### 2.4. Experimental Design

**Figure 2:**
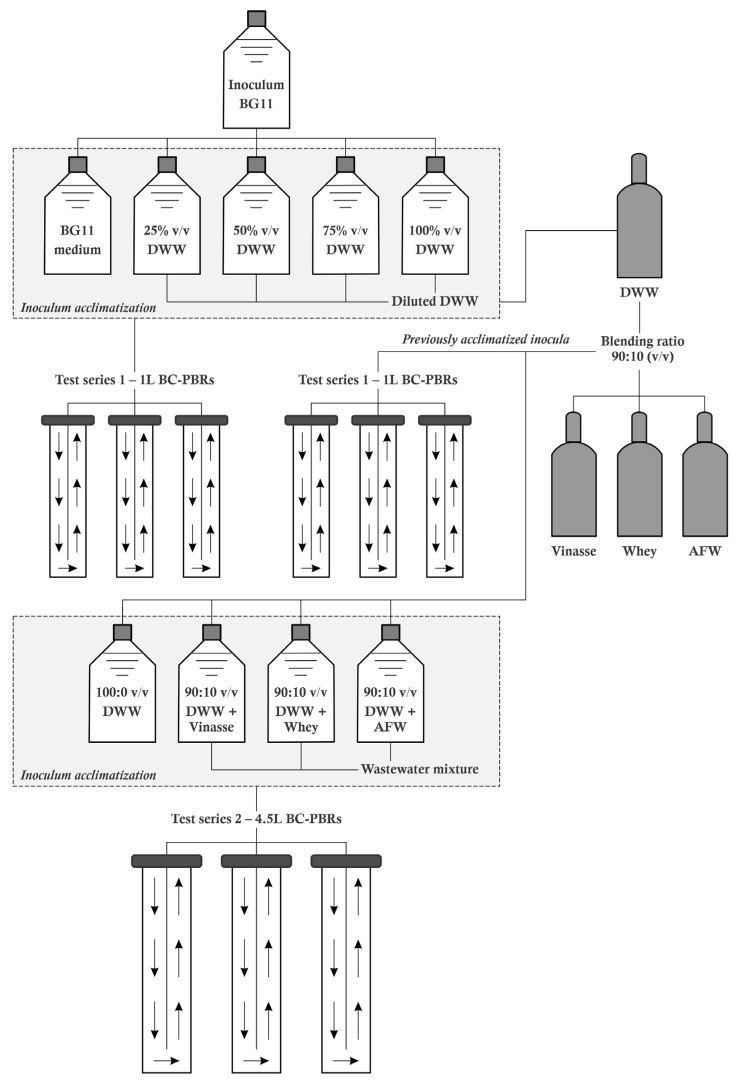
Schematic representation of the experimental system.

Figure 2 presents a schematic of the experimental setup. Two different configurations were employed, each tailored to the specific test series, with three BC-PBR units in each configuration.

#### 2.4.1. The 1 L BC-PBRs Culture Experiments

Initially, the tolerance of *Chlorella* sp. MC18 and *Scenedesmus* sp. MJ23-R to different DWW formulations was evaluated in 1 L BC-PBRs. Treatments consisted of various concentrations of DWW diluted with distilled water (25%, 50%, 75%, and 100% *v*/*v*). Standard BG11 medium was used as the control culture. Subsequently, the previously selected DWW proportion (i.e., 100% or 100:0 *v*/*v*) was employed to maximize biomass production. Cells of each microalga were cultivated in mixotrophic mode in DWW supplemented with vinasse, whey, and AFW at a concentration of 90:10 (*v*/*v*). Previous studies have shown that a 90:10 (*v*/*v*) blending ratio of a medium or diluent with vinasse, whey, or AFW ensures optimal microalgal growth kinetics compared to other dilutions, with minimal interference from heterotrophs [5,6,29]. The initial inoculum density for each culture was set at 1.0 × 10^6^ cells mL^−1^. For both treatment and control cultures, the pH was adjusted to 7.0 ± 0.2 using a 5 M KOH solution prior to inoculation to optimize growth and maximize photosynthetic activity of *Chlorella* and *Scenedesmus*. The incubation period was eight days, and cultivation conditions and photobioreactor operational parameters followed those described in Section 2.1 and Section 2.3. All experiments were conducted in triplicate, and each medium was sterilized by microfiltration through a 0.22 µm PTFE membrane. During cultivation in the 1 L BC-PBRs, daily measurements of cell density and optical density at 570 nm (OD_570_) and 683 nm (OD_683_) were performed.

#### 2.4.2. The 4.5 L BC-PBRs Culture Experiments

In the following set of experiments, the batch treatment cultures (i.e., the wastewater blend cultures) were scaled up in a 4.5 L BC-PBR following the principle of geometric similarity. Using an initial inoculum concentration of 10 mg_DW_ L^−1^, *Chlorella* sp. and *Scenedesmus* sp. were cultivated for eight days. Specifically, the PAR irradiance was 120 μmol photons m^−2^ s^−1^, the temperature was maintained at 25 ± 2 °C, the atmospheric CO_2_ flow rate was 8 L min^−1^ (i.e., 0.5 vvm), and the light/dark (L/D) cycle was 12:12 h. All experiments were performed in triplicate. The installation of a top-mounted sampling port in the BC-PBR allowed intermittent analysis of dry biomass and biochemical lipid assays. Additionally, at the beginning and end of each treatment, the chemical composition of each medium formulation based on the DWW blend with vinasse, whey, and AFW (90:10 *v*/*v*, respectively) was analyzed.

### 2.5. Microalgal Growth Monitoring

The growth of each algal species was monitored using two analytical methods: (i) cell concentration (*X_C_*, ×10^6^ cells mL^−1^), determined by counting cells in a hemocytometer (Marienfeld, Lauda-Königshofen, Germany) under an optical microscope (Carl Zeiss, Primo Star, Göttingen, Germany) at 40× magnification; and (ii) optical density (OD, arbitrary units, AU), measured as absorbance at 570 nm and 683 nm using a UV–Vis spectrophotometer (UH-5300, Hitachi, Hitachinaka, Japan), with sterile distilled water as the blank. Measurement of OD is widely considered as a rapid and cost-effective method to estimate microalgal cell or biomass concentrations in photobioreactors [30]. Linear correlations between these two response variables (optical density and cell concentration) in DWW and BG11 treatment cultures were generated using GraphPad Prism 9.0.2. software.

Biomass production was monitored by determining the dry weight concentration (*X_B_*, mg_DW_ L^−1^). Dry biomass was measured by centrifuging 20 mL of each microalgal culture at 15,025 RCF-max for 10 min at 4 °C. To remove residual medium, the pellets were washed three times with sterile distilled water. The resulting biomass was then dried in a hot-air oven at 105 °C for 24 h until constant weight was achieved, and finally weighed using a high-precision balance (AS 220.R2 PLUS, RADWAG, Radom, Poland).

Growth kinetics—including the maximum specific growth rate (*μ_max_*, d^−1^), biomass yield (*Y_B_*, mg L^−1^), and biomass productivity (*P_B_*, mg L^−1^ d^−1^)—for batch experiments with *Chlorella* sp. MC18 and *Scenedesmus* sp. MJ23-R were calculated using Equations (1)–(3) according to Mamani et al. [29].

*µ_max_* (d^−1^) = (ln *X* − ln *X*_0_)/Δ*t* (1)

*Y_B_* (mg L^−1^) = *X_max_* − *X*_0_ (2)

*P_B_* (mg L^−1^ d^−1^) = (*X_max_* − *X*_0_)/Δ*t* (3)

where *X* represents the cell concentration at a specific culture time (*t*), and *X_max_* and *X*_0_ are the maximum and initial cell concentration, respectively. Δ*t* is interval of time (in days) between *X* and *X*_0_ or *X_max_* and *X*_0_.

### 2.6. Analytical Measurements

The physical parameters, namely pH and electrolytic conductivity (EC) were measured using a HI98130 meter (HANNA Instruments, Padova, Italy). Color and chemical parameters—including total suspended solids (TSS), chemical oxygen demand (COD), total nitrogen (TN), and total phosphorus (TP)—were analyzed following the analytical methods prescribed in SMEWW–APHA–AWWA–WEF, 24th Ed., 2023. The specific method for each analysis is detailed in Rice et al. [31]: color (Part 2120 C); TSS (Part 2540 D); COD (Part 5220 D); TN (Part 4500-N C); and TP (Part 4500-P E).

Micronutrient quantification—including boron (B), calcium (Ca), cobalt (Co), copper (Cu), iron (Fe), lithium (Li), magnesium (Mg), manganese (Mn), molybdenum (Mo), nickel (Ni), potassium (K), silicon (Si), sodium (Na), and zinc (Zn)—was performed using inductively coupled plasma mass spectrometry (ICP-MS) for selected elements, including uranium isotopes (see ISO 17294-2 [32] for details).

The removal efficiencies of macro- and micronutrients (*REi*, %) in 4.5 L BC-PBR batch experiments were calculated according to Equation (4):

*REi* (%) = (C_0_ − C*_f_*)/C_0_ × 100 (4)

where C_0_ and C*_f_* represent the concentration of the specific nutrient (*i*) at the beginning and at the end of the experiment, respectively.

### 2.7. Lipid Extraction from Microalgal Cells

The extraction and concentration of lipids (mg L^−1^) of *Chlorella* sp. MC18 and *Scenedesmus* sp. MJ23-R was determined using the EPA Method 1664—Review B [33]. This analysis was conducted by an external laboratory (http://www.cerper.com, Lima, Peru) (accessed on 5 November 2025), accredited by the National Quality Institute and certified under the international ISO 9001:2015 standard (https://www.iso.org/standard/62085.html, Geneva, Switzerland) (accessed on 5 November 2025), thereby ensuring the authenticity, reliability, and representativeness of the results. Briefly, 1000 mL of culture broth was centrifuged in a refrigerated high-speed centrifuge (15,025 RCF-max for 10 min at 4 °C), and the biomass pellets were gently washed three times with sterile deionized water, discarding the supernatants after each wash. The resulting biomass was resuspended in 1 L of ultrapure water (Milli-Q, 18.2 MΩ·cm) and acidified with 2500 µL of 6 M H_2_SO_4_ to reach pH ≤ 2, thereby lysing the microalgal cells to release intracellular lipids. The lipids were then extracted with n-hexane (95% purity) in a separatory funnel, with a standard sequence of three serial extractions. The hexane-extractable material (HEM) was subsequently dried using a rotary evaporator and analyzed gravimetrically. Cheng et al. [34] confirm that lipid extraction using hexane in microalgal samples is a highly efficient method in terms of yield and applicability. The lipid content was additionally quantified as a percentage of the biomass dry weight (% DW).

Lipid yield (mg L^−1^) and lipid productivity (mg L^−1^ d^−1^) for both microalgal species were calculated according to Equations (5) and (6), respectively.

Lipid yield (mg L^−1^) = *L_N_* − *L*_0_ (5)

Lipid productivity (mg L^−1^ d^−1^) = (*L_N_* − *L*_0_)/Δ*t* (6)

where *L_N_* represents the lipid concentration at a given cultivation time (*t*), while *L*_0_ represents the initial lipid concentration. Δ*t* is interval of time (in days) between *L_N_* and *L*_0_.

### 2.8. Statistical Analysis

All batch experiments were performed in triplicate (*n* = 3), and data are presented as mean ± standard deviation (±SD). The assumptions of normality and homoscedasticity were tested prior to conducting one-way ANOVA of the treatment cultures, using IBM SPSS Statistics 29.0.2.0 (SPSS Inc., Chicago, IL, USA). Tukey’s honestly significant difference (HSD) test was applied to identify significant differences (*p* < 0.05) between groups.

## 3. Results and Discussion

### 3.1. Effects of DWW Culture Media on Microalgae Growth Kinetics

**Figure 3:**
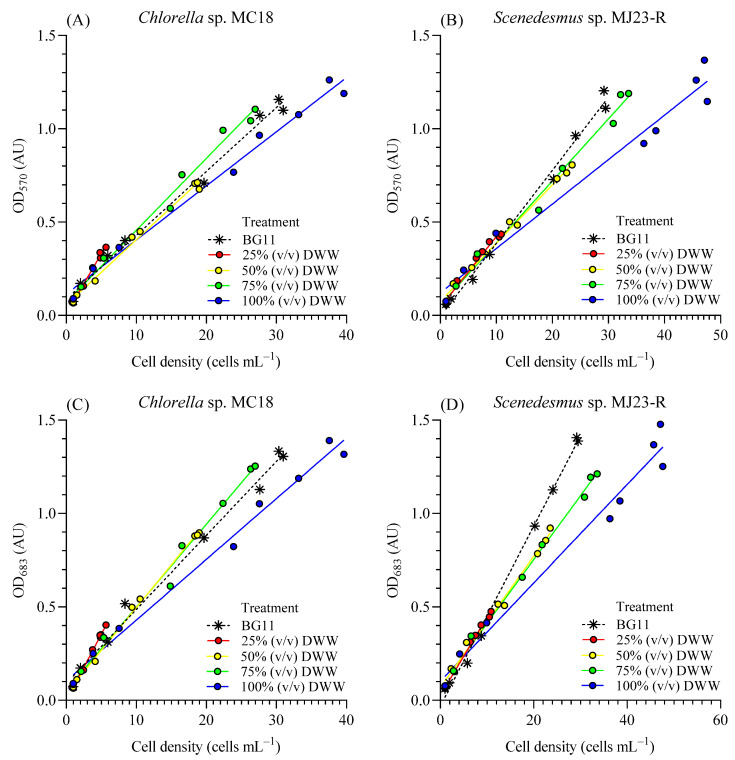
Standard curve between optical density at 570 nm (OD_570_, AU) and 683 nm (OD_683_, AU) and cell density (×10^6^ cell mL^−1^) for *Chlorella* sp. MC18 (**A**,**C**) and *Scenedesmus* sp. MJ23-R (**B**,**D**) grown in 1 L BC-PBRs in varying concentrations of domestic wastewater (DWW: 25%, 50%, 75% and 100%, *v*/*v*) and inorganic control (BG11). Mean ± SD based on *n* = 3 culture replicates.

Figure 3A–D show positive correlations between cell density and optical density for *Chlorella* sp. and *Scenedesmus* sp. The OD_570_ reflects particulate matter or turbidity concentration in the medium, encompassing both microalgal cells and non-photosynthetic particles [35]—that is, microorganisms or heterotrophs that may remain in DWW despite filtration with a 0.22 µm PTFE membrane. OD_683_ corresponds to a specific absorption peak of chlorophyll in *Chlorella* sp. and *Scenedesmus* sp. [36]. The strong positive correlation (R^2^ > 0.98) between cell concentration (*X_C_*, cells mL^−1^) and optical density at both wavelengths (570 nm and 683 nm) across all treatments suggests that most of the detected particles correspond to *Chlorella* sp. or *Scenedesmus* sp. cells.

In addition, possible heterotrophs (e.g., bacteria, viruses, and others present in DWW) are much smaller than microalgal cells, which likely results in minimal detection by absorbance. As also reported by Condori et al. [8], microscopic observations confirmed these findings, showing differences in cell sizes among treatments and lower variability within individual cultures. In line with this, microalgae such as *Chlorella vulgaris* can be effectively monitored via spectrophotometry due to their larger size and pigment content. Almomani and Örmeci [37] emphasized that smaller organisms or those with lower pigmentation, such as many bacteria, are difficult to detect using absorbance-based methods because of weaker signals and higher background interference. Recently, Cartin-Caballero et al. [38] described how differential sedimentation and optical density approaches exploit size and pigment contrasts between microalgae and methanotrophic bacteria to enhance detection. For instance, microalgae such as *Galdieria* sp. are larger and more pigmented than bacteria like *Methylacidiphilum* sp., which improves absorbance-based quantification. These results confirm that the smaller size and lower pigment content of bacteria lead to weaker absorbance signals compared to microalgae, and that optical density is a reliable method for determining cell density in microalgal cultures.

**Figure 4:**
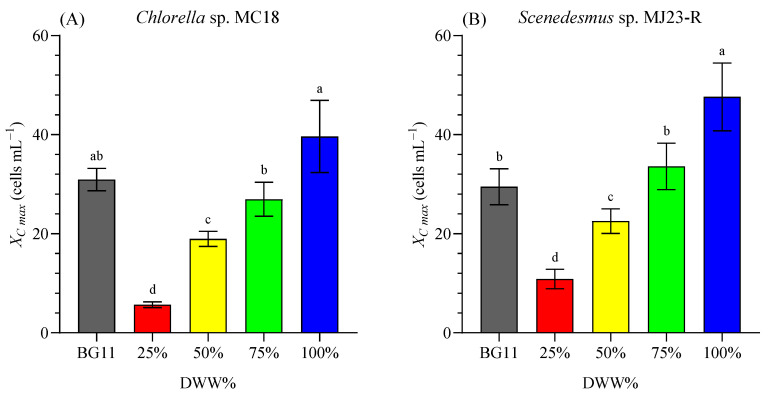
Maximum cell concentration (*X_C max_*, in ×10^6^ cell mL^−1^) of *Chlorella* sp. MC18 (**A**) and *Scenedesmus* sp. MJ23-R (**B**) grown in 1 L BC-PBRs in varying concentrations of domestic wastewater (DWW: 25%, 50%, 75% and 100%, *v*/*v*) and inorganic control (BG11). Mean ± SD based on *n* = 3 culture replicates. Data indexed with different letters indicate statistically significant differences between treatments ((**A**): *p* < 0.001; (**B**): *p* < 0.001) according to one-way ANOVA analysis.

Figure 4A,B show a similar performance of both microalgae exposed to increasing concentrations of DWW diluted with distilled water (25%, 50%, 75%, and 100%). The highest cell density for CH 39.63 ± 7.28 (×10^6^ cells mL^−1^), and SC 47.63 ± 6.84 (×10^6^ cells mL^−1^) was reached in the treatments with 100% DWW. In addition, the COD (mg L^−1^) of DWW was reduced by 24% in *Chlorella* sp. culture and by 7% in *Scenedesmus* sp. culture. The COD removal efficiency (*REi*, %) was 76.2%, and 93.1% after 8 days of cultivation of *Chlorella* sp. and *Scenedesmus* sp., respectively. Similarly, Dang et al. [39] obtained high COD removal efficiency (exceeding 90%). Microalgal assimilation, followed by bacterial denitrification, was the major pathway of removing total nitrogen when the C/N ratio exceeded 5:1., highlighting that the use of phycosphere-associated bacteria could be a promising strategy for controlling nutrient pollution in wastewater [39].

Based on these results, it was observed that among the treatments where DWW was diluted with freshwater or distilled water (25–100%), the best performance occurred in the 100% DWW condition. Notably, growth was comparable (*p* ≥ 0.05) for *Chlorella* sp. and even significantly higher (*p* < 0.05) for *Scenedesmus* sp. than in the control medium (BG11). BG11 is a nutrient-rich formulation widely used for cultivating freshwater cyanobacteria and microalgae, containing multiple inorganic salts and trace metals, which contributes to its high nutritional value but also to its higher cost compared with simpler media [40].

In this study, identifying one or more media that outperforms BG11 is highly relevant for reducing cultivation costs and enhancing the sustainability of the algal bioprocess. To evaluate the tolerance of both microalgae to DWW, it was initially diluted at various proportions (25–75%). However, the maximum concentration (100%) supported the best growth performance of *Chlorella* sp. and *Scenedesmus* sp. Therefore, 100% DWW was selected for subsequent experiments due to its minimal environmental impact and optimal economic considerations. It should be noted that the DWW did not undergo dilution, nor complex or costly pretreatment processes, but only sedimentation by gravity. Moreover, using the maximum concentration of DWW significantly reduces the water footprint of the process by avoiding the need for additional freshwater. In this line, Schmuck et al. [25] also used 100% urban wastewater to assess the bioremediation potential of *Chlorella vulgaris*, and simultaneously produce value-added biomass (proteins, carbohydrates, lipids, and fatty acids).

**Figure 5:**
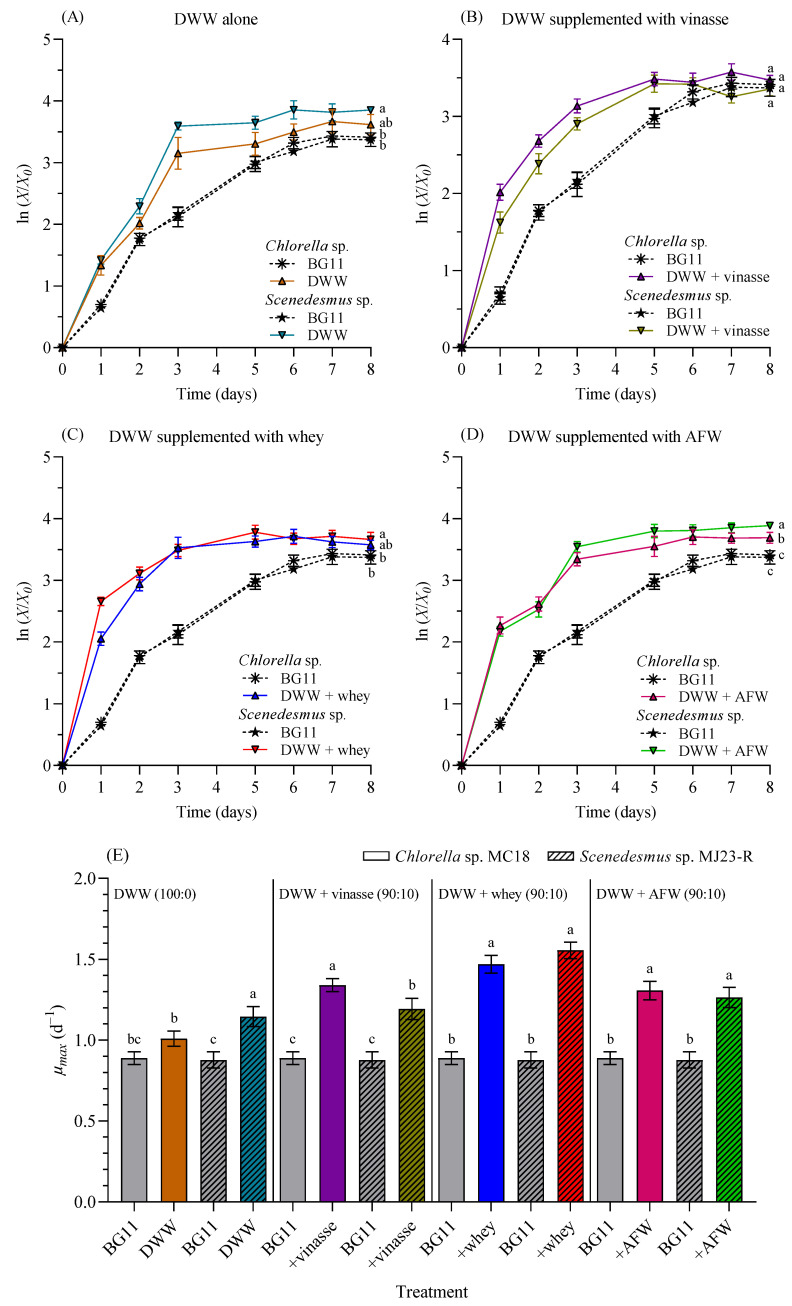
Log-normalized growth profiles (*X*/*X*_0_) of *Chlorella* sp. and *Scenedesmus* sp. grown in 1 L BC-PBRs in domestic wastewater (DWW, 100:0 *v*/*v*) (**A**) and in different formulations of DWW mixtures with vinasse (**B**), whey (**C**), and agro-food waste (AFW) (**D**) within a 90:10 (*v*/*v*) range. (**E**) Maximum specific growth rate (*µ_max_*, d^−1^). Control as BG11 medium. Mean ± SD based on *n* = 3 culture replicates. Data indexed with different letters indicate statistically significant differences between treatments ((**A**): *p* = 0.002; (**B**): *p* = 0.366; (**C**): *p* = 0.018; (**D**): *p* < 0.001; (**E**): *p* ≤ 0.001) according to one-way ANOVA analysis.

Figure 5 presents the log-normalized growth profiles of *Chlorella* sp. and *Scenedesmus* sp. cultivated in 1 L BC-PBRs with 100% DWW (Figure 5A), as well as in different formulations of DWW mixed with distillery wastewater (vinasse) (Figure 5B), dairy wastewater (whey) (Figure 5C), and agro-food waste (AFW) (Figure 5D) at a 90:10 (*v*/*v*) ratio. Figure 5E compares the maximum specific growth rates (*µ_max_*, day^−1^) of both microalgae in BG11 medium, 100% DWW, and DWW combined at 90:10 (*v*/*v*) with vinasse, whey, and AFW, respectively.

The *µ_max_* in 100% DWW was significantly higher (*p* < 0.05) than in BG11 medium for *Scenedesmus* sp. (1.145 ± 0.062 d^−1^), but not for *Chlorella* sp. (1.009 ± 0.047 d^−1^). Supplementation of DWW with 10% vinasse resulted in significantly higher growth rates compared to BG11 medium for both *Chlorella* sp. (1.340 ± 0.039 d^−1^), and *Scenedesmus* sp. (1.192 ± 0.065 d^−1^) (*p* < 0.05). Similarly, when DWW was supplemented with 10% whey, growth rates were significantly higher (*p* < 0.05) than in BG11 for both microalgae (1.469 ± 0.054 d^−1^) and (1.556 ± 0.051 d^−1^), respectively. The same pattern was observed when DWW was supplemented with 10% AFW (1.307 ± 0.058 d^−1^) and (1.264 ± 0.062 d^−1^), for both algae strains.

As previously demonstrated (Figure 4), the optimal proportion for maximizing the reproduction of *Chlorella* sp. and *Scenedesmus* sp. was 100% DWW. However, this medium lacks certain nutrients or contains them at relatively low concentrations (see Table 1), preventing it from becoming an ideal medium for microalgal cultivation. Typically, when a waste stream lacks essential nutrients, supplementation with chemical nutrients, or blending with chemically defined media (e.g., BG11 or Bold’s basal medium) is required. Nevertheless, this strategy increases costs due to the addition of chemicals, and prevents the reuse of polluting effluents. In this study, DWW-based cultures (100% or 100:0 *v*/*v*) were not supplemented with inorganic media nutrients but rather with other wastewaters (vinasse, whey, or agro-food waste), which provided a wide variety of macro- and micro-nutrients (see Table 1). This approach significantly improved culture performance (expressed as higher *µ_max_*) for both microalgae (Figure 5).

Experimental data revealed that supplementation with 10% vinasse, whey, or AFW further increased *µ_max_* compared to 100% DWW. In fact, under these supplementation conditions, the cultures significantly outperformed the control cultures grown in BG11 medium. Both microalgal species exhibited similar growth patterns. The micronutrients and minerals provided by the supplementary media (see Table 1) were adequate to improve the growth of CH and SC. The overall performance of both microalgae was additionally tested by other final points (e.g., biomass yield, biomass productivity, and lipid yields), and will be further discussed later.

When a growth medium is supplemented with organic carbon, microalgae can adopt a mixotrophic metabolism, providing greater opportunities to regulate and optimize growth and development. In the present study, vinasse, whey, and agro-food wastewater were selected specifically for their high organic load. It is noteworthy that organic matter—commonly expressed as chemical oxygen demand (COD)—is a key factor in enhancing microalgal yield under heterotrophic conditions. In this regard, Rasheed et al. [41] reported that the presence of organic matter in mixed culture media (such as DWW) can significantly improve microalgal growth by supplying additional sources of carbon and energy, consistent with the findings of the present study, where DWW supplemented with three different types of wastewaters promoted enhanced performance. Organic matter, measured as COD, can thus be a key factor in improving microalgal culture performance, especially under conditions that are not strictly photoautotrophic (e.g., heterotrophic or mixotrophic) [26].

In a review on mixotrophic and heterotrophic growth of microalgae using acetate derived from various production processes, Proietti et al. [42] highlighted the high metabolic flexibility of microalgae, noting that mixotrophic cultivation—combining light with organic carbon sources such as acetate—can significantly enhance biomass productivity while reducing environmental impact. This metabolic flexibility enables tailored cultivation strategies depending on available resources and desired outcomes.

### 3.2. Process Scaling-Up in 4.5 L Photobioreactors

Subsequently, the scale-up of the process in 4.5 L photobioreactors with a 4.0 L working volume was carried out. Bubble column photobioreactors (BC-PBRs) offer simple construction, efficient mass transfer with low energy consumption, and ease of long-term sterile operation. Merchuk [43] and Machado-Cepeda et al. [44] highlighted that BC-PBRs are attractive due to their vertical design, gas bubbling system for mixing, and ease of scaling-up. These reactors are noted for their simple construction and effective gas–liquid mass transfer, which supports high-yield microalgal cultivation with minimal energy input. Their design eliminates the need for mechanical agitation, reducing energy consumption and simplifying sterilization and maintenance over long-term operation. Under the biorefinery concept, Machado-Cepeda et al. [44] compile the most recent information about configuration parameters in bubble column photobioreactors for obtaining phycocyanin and protein from cyanobacterial and microalgal biomass, as a source of high added-value compounds such as proteins, pigments, carbohydrates, and lipids, among others, for different industry applications.

**Figure 6:**
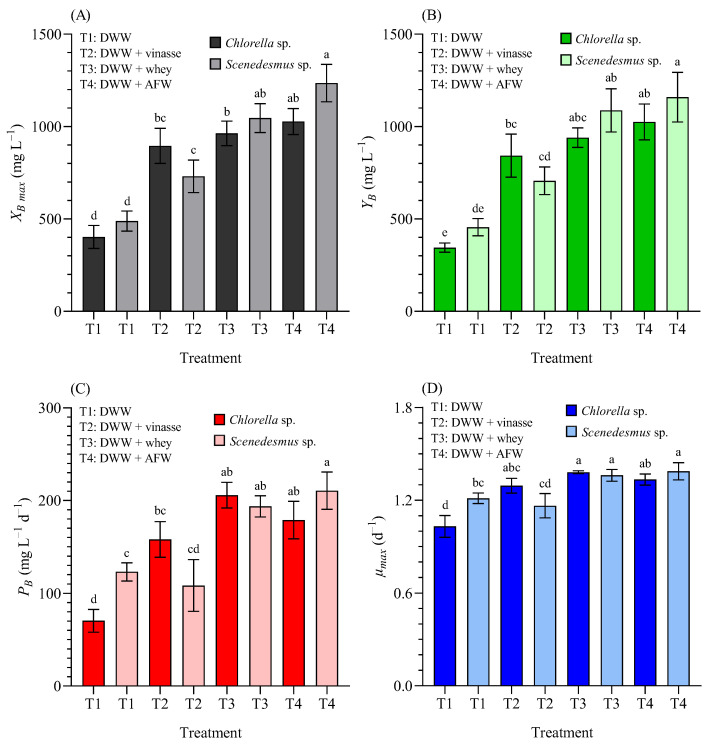
(**A**) Maximum biomass concentration (*X_B max_*, mg L^−1^), (**B**) biomass yield (*Y_B_*, mg L^−1^), (**C**) biomass productivity (*P_B_*, mg L^−1^ d^−1^), and (**D**) maximum specific growth rate (*µ_max_*, d^−1^) of *Chlorella* sp. and *Scenedesmus* sp. grown in 4.5 L BC-PBRs in domestic wastewater (DWW, 100:0 *v*/*v*) and in mixture formulations with vinasse, whey, and agro-food waste (AFW) within a 90:10 (*v*/*v*) range. Mean ± SD based on *n* = 3 culture replicates. Data indexed with different letters indicate statistically significant differences between treatments ((**A**): *p* < 0.001; (**B**): *p* < 0.001; (**C**): *p* < 0.001; (**D**): *p* < 0.001) according to one-way ANOVA analysis.

In this study, Figure 6 illustrates the growth kinetics of *Chlorella* sp. (CH) and *Scenedesmus* sp. (SC) in terms of biomass concentration on a dry weight (DW) basis.

(Figure 6A) shows the maximum biomass concentration (*X_B max_*); (Figure 6B) the biomass yield (*Y_B_*); (Figure 6C) the biomass productivity (*P_B_*); and (Figure 6D) the maximum specific growth rate (*µ_max_*), of *Chlorella* sp. and *Scenedesmus* sp. grown in 4.5 L BC-PBRs in domestic wastewater (DWW, 100:0 *v*/*v*) and in different formulations of DWW mixtures with vinasse, whey, and agro-food waste, within a 90:10 (*v*/*v*) concentration.

The maximum biomass concentration (*X_B max_*) significantly increased in blended media compared with 100% DWW for both microalgae (*p* < 0.05) (Figure 6A). In CH cultures, the *X_B_* was >403, >895, >963, >1027 mg_DW_ L^−1^ in 100% DWW, and supplemented with 10% vinasse, whey and AFW, respectively. In SC cultures, the *X_B_* was >489, >731, >1046, and >1235 mg_DW_ L^−1^ in 100% DWW, and supplemented with 10% vinasse, whey and AFW, respectively.

The *Y_B_* also increased in DWW blended media: from 344.7 ± 25.2 mg_DW_ L^−1^ (CH), and 455.3 ± 46.8 mg_DW_ L^−1^ (SC) to >843, >940, >1025, (CH), and >707, >1087, >1159 mg_DW_ L^−1^ (SC) in DWW and supplemented with vinasse, whey and AFW, respectively (Figure 6B). The *P_B_* increased from 70.46 ± 12.29 mg_DW_ L^−1^ d^−1^ (CH) and 123.11 ± 9.72 mg_DW_ L^−1^ d^−1^ (SC) in 100% DWW, to 158.01 ± 19.28, 205.79 ± 13.87, 178.90 ± 20.24 mg_DW_ L^−1^ d^−1^ (CH), and 108.44 ± 27.75, 193.78 ± 11.48, and 210.67 ± 20.12 mg_DW_ L^−1^ d^−1^ (SC) in DWW supplemented with 10% vinasse, whey and AFW, respectively (Figure 6C). Finally, in DWW, the *µ_max_* (day^−1^) was significantly higher in SC than in CH. In blended media, whey and AFW significantly enhanced *µ_max_* (*p* < 0.05) for both microalgae (Figure 6D).

El-Sheekh et al. [13] studied the effect of wastewater on the growth rate of three green microalgae using the density of algal cells (measured as optical density, OD_560_) and cellular dry weight (CDW) (biomass productivity) after 10 days of incubation. The best performance was achieved by *Chlamydomonas reinhardtii* showing the highest biomass productivity in wastewater (48.62 mg L^−1^ day^−1^) corresponding to 40.2 mg L^−1^ day^−1^, and *Monoraphidium braunii* had the highest lipid content and lipid productivity, which was more than twofold higher to the synthetic medium (control). The author attributes these results to the higher organic matter, and phosphorus available in sewage wastewater.

**Figure 7:**
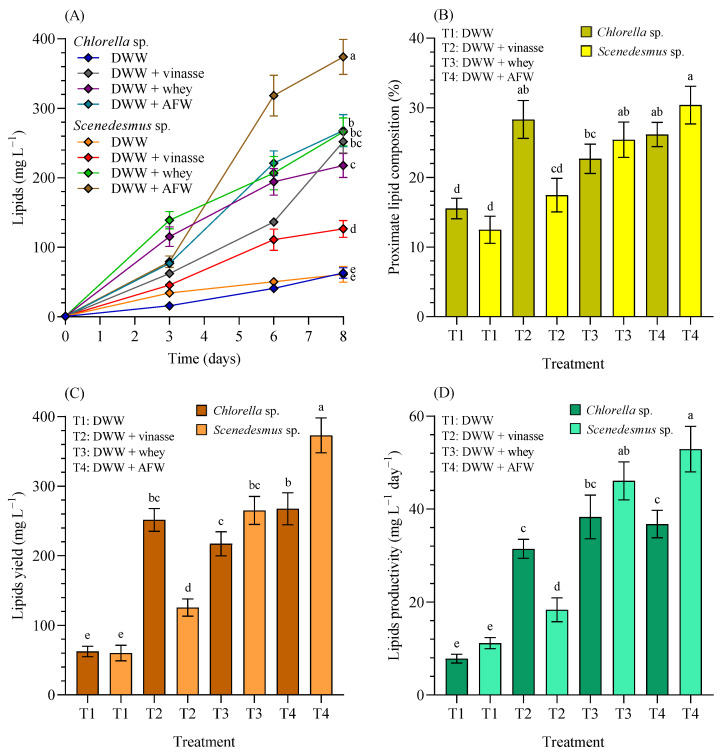
(**A**) Lipid production (mg L^−1^), (**B**) proximate lipid concentrations in percentage of dry weight (% dw), (**C**) lipid yield (mg L^−1^), and (**D**) lipid productivity (mg L^−1^ d^−1^) of *Chlorella* sp. and *Scenedesmus* sp. grown in 4.5 L BC-PBRs in domestic wastewater (DWW, 100:0 *v*/*v*) and in mixture formulations with vinasse, whey, and agro-food waste (AFW) within a 90:10 (*v*/*v*) range. Mean ± SD based on *n* = 3 culture replicates. Data indexed with different letters indicate statistically significant differences between treatments ((**A**): *p* < 0.001; (**B**): *p* < 0.001; (**C**): *p* < 0.001; (**D**): *p* < 0.001) according to one-way ANOVA analysis.

Figure 7A shows the lipid production; Figure 7B the proximate lipid concentrations in percentage of dry weight (% dw); Figure 7C the lipid yield, and Figure 7D the lipid productivity of *Chlorella* sp. and *Scenedesmus* sp. grown in 4.5 L BC-PBRs in domestic wastewater (DWW, 100:0 *v*/*v*) and in different formulations of DWW mixtures with vinasse, whey, and agro-food waste within a 90:10 (*v*/*v*) range.

As previously indicated, the main objective of this work was to maximize biomass production, specifically lipids. According to Figure 7A, when cultivated with 100% DWW, *Chlorella* sp. or *Scenedesmus* sp. were unable to achieve high lipid content (63.03 ± 7.56 and 61.07 ± 11.30 mg L^−1^) without significant differences between them (*p* > 0.05). However, when supplemented with vinasse, whey, or AFW, lipid production increased significantly (*p* < 0.05) in all the treatments compared with DWW, with maximum lipid concentration achieved in DWW + AFW for CH (268.07 ± 22.95 mg L^−1^), and also for SC (373.97 ± 25.19 mg L^−1^). The proximate lipid composition (%) (Figure 7B) in 100% DWW reached 15.54 ± 1.48% (CH) and 12.49 ± 1.94% (SC). After the supplementation increased to 28.33 ± 2.72%, 22.69 ± 2.11% and 26.17 ± 1.75% (CH), and 17.46 ± 2.41%, 25.42 ± 2.53% and 30.40 ± 2.70% (SC) in DWW supplemented with 10% vinasse, whey and AFW, respectively.

The lipid yield (Figure 7C): in CH with 100% DWW reached 62.50 ± 7.54 mg L^−1^, notably increasing to 251.43 ± 16.38, 217.10 ± 17.28, and 268.53 ± 22.95 mg L^−1^; and 60.23 ± 11.29 mg L^−1^ in SC with 100% DWW, increasing to 125.70 ± 12.36, 265.13 ± 20.03, and 373.13 ± 25.15 mg L^−1^ in DWW cultures, and after the supplementation with 10% vinasse, whey and AFW, respectively. Finally, considering the lipid productivity (Figure 7D), CH with 100% DWW was 7.81 ± 0.94 mg L^−1^ d^−1^, and SC with 100% DWW was 11.16 ± 1.20 mg L^−1^ d^−1^. However, after supplementation, it increased to 31.43 ± 2.05, 38.29 ± 4.70, and 36.77 ± 2.95 mg L^−1^ d^−1^ (CH), while SC also increased the lipid productivity to 18.34 ± 2.57, 46.07 ± 4.09, and 52.90 ± 4.89 mg L^−1^ d^−1^ after the supplementation with 10% vinasse, whey and AFW, respectively. In brief, lipid production kinetics significantly increased in blended media compared to DWW for both microalgae (*p* < 0.05). This study standardized a mechanism to maximize lipid concentration, which could serve as a feedstock for various biotechnological applications (e.g., biofuels). However, further research is required to characterize the extracted lipids and to assess their commercial potential.

The potential of *C. vulgaris* to bioremediate urban wastewater and to jointly produce value-added biomass was studied by Schmuck et al. [25]. After 8 days, *C. vulgaris* produced 90.1% fatty acids, primarily oleic acid and polyunsaturated fatty acids, lipid increased 1.9-folds than in synthetic culture, surpassing reported values in the literature. This makes it attractive for the food industry, among others, due to the antioxidant properties that PUFAs offer, and its omega-3 and omega-6 content further enhances its attractiveness for various industries, including the pharmaceutical, therapeutic, and nutraceutical sectors.

Lipid accumulation in microalgae is associated with various types of stress (e.g., high organic carbon load, nutrient limitation, salinity stress, among others). Mercado et al. [45] showed that *Scenedesmus* sp. cultivated in dairy wastewater enhanced lipid accumulation (507.81 ± 19.09 mg g^−1^) compared to standard media. The stress from organic load and nutrient imbalance in the effluent triggered lipid biosynthesis while maintaining growth. In turn, the study by Gour et al. [46] evaluated the effect of salinity stress on *Scenedesmus quadricauda*, *Scenedesmus dimorphus*, and *Chlorella* sp. Lipid content increased significantly under 160 mM NaCl stress, reaching up to 39.42% in *Scenedesmus dimorphus* and 32.19% in *Chlorella* sp. The authors concluded that stepwise salinity stress enhances lipid productivity without severely compromising growth. Sun et al. [47] discussed how to resolve the conflict between cell growth and the production of valuable molecules, proposing two-stage cultivation strategy, dedicating the first stage with optimum growth conditions to gain the maximum biomass production, while reserving the second process for the accumulation of lipids or carotenoids under various stress conditions, or supplementation with growth-promoting agents like phyto-hormones. For example, exogenous addition of abscisic acid (ABA) increased the dry biomass yield up to 2.1-fold of *S. quadricauda* compared to nitrogen-deficient cells, respectively [48].

Suparmaniam et al. [49] review that exposing microalgae to abiotic stress environments—such as nutrient starvation, high salinity, and strong light intensity—can trigger cellular mechanisms that elevate lipid accumulation by redirecting metabolic pathways. Interestingly, Alkhamis et al. [50] found that pH-induced stress significantly increased lipid productivity during the starvation phase, confirming stress as a driver of lipid biosynthesis. Conversely, in our work *Chlorella* sp. and *Scenedesmus* sp. increased pH from 6.8 (DWW) to 8.39–8.57 with DWW + vinasse; 8.00–8.46 under whey-supplemented treatment, and 7.75–7.98 under AFW supplementation in *Chlorella* sp. and *Scenedesmus* sp. cultures, respectively. As can be seen, in this study, supplementation enhanced pH conditions, stabilizing cultures in alkaline values, optimal growing conditions for these microalgae (see Table 2).

A proper carbon/nitrogen (C/N) ratio operation might facilitate microalgae metabolism and the ability to effectively absorb nitrogen and phosphate from wastewater. Research on microalgae has shown that they tend to accumulate high levels of lipids when exposed to various stress conditions [12]. For instance, a high C/N ratio leads to increased lipid content [51]. Limited nitrogen availability restricts growth kinetics and protein synthesis; however, this condition encourages *Chlorella* or *Scenedesmus* to store excess organic carbon as intracellular lipids. In this study, the C/N ratio and the improved nutrient profile were the main causes driving lipid accumulation. Previous to the treatment with microalgae, the C/N was 6.5, 18.5, 76.2 and 122.3 for the DWW, DWW + vinasse, DWW + whey and DWW + AFW, respectively, showing a clear increase in the C/N relation in the different blended culture media compared with DWW alone. Elevated C/N ratios suggest that nitrogen availability may be sufficient to support microalgal growth, aligning with Zhu et al. [52], who noted that municipal wastewater with C/N ratios of 3:1 to 4:1 might be inadequate for complete nitrogen assimilation by microalgal biomass without supplementation. Similarly, Dang et al. [39] observed enhanced biomass productivity at a C/N ratio of 5:1, reaching 106 mg L^−1^ d^−1^. Zhu et al. [52] further demonstrated that a co-culture system of *Chlorella vulgaris* and activated sludge outperformed the sludge-only system, achieving a biomass productivity of 343.3 mg L^−1^ d^−1^. This high C/N ratio indicates that the available N could be enough to microalgae growth, considering Zhu et al. [52] suggestion that municipal wastewater having C/N ratios around 3:1 to 4:1 may be too low to support all the nitrogen assimilation by microalgae biomass, unless supplemented.

Under the “blending scenario”, this study found that DWW supplementation with various wastewaters led to significant increases in biomass yield and lipid productivity, confirming the regulatory potential of mixotrophic metabolism. This approach opens new opportunities for biomass optimization in a cost-effective, pragmatic, and environmentally sustainable way. A similar approach was proposed by Anagnostopoulou et al. [10], who cultivated *C. vulgaris*, *Nannochloropsis oculata*, and *Scenedesmus* sp. using a waste mixture (brewery effluent, cheese whey, and expired orange juice) as a nutrient source. They proposed this as a low-cost, low-CO_2_, and circular solution that mitigates eutrophication pressure while substituting synthetic fertilizers. As in our study, the authors specifically emphasized its potential for generating lipid-rich biomass for value-added applications.

### 3.3. Comparison of Physicochemical Composition Before and After Culture Supplementations

Table 2 shows the physicochemical composition of DWW, 100:0 *v*/*v* and DWW mixture formulations with distillery wastewater (vinasse), dairy wastewater (whey) and agro-food waste (AFW) (90:10 *v*/*v*, respectively), before and after the cultivation of *Chlorella* sp. (CH) and *Scenedesmus* sp. (SC), and in Table 3, the macronutrient and micronutrient removal efficiency (*REi*, %) after cultivation of CH, and SC, in domestic wastewater (DWW, 100:0 *v*/*v*) and in different formulations of DWW mixtures (90:10 *v*/*v*), can be observed.

#### 3.3.1. Removal of Organic Matter and Nutrients

After supplementation, the wastewater showed a marked increase in organic and nutrient loads compared to DWW alone (see Table 2). COD levels rose dramatically—by 292-, 269-, and 314-fold in DWW blended with vinasse, whey, and AFW, respectively. Similarly, total nitrogen increased 92.9-, 13.1-, and 6.8-fold, while total phosphorus rose 48-, 167.6-, and 18.5-fold, respectively, indicating a substantial enrichment of organic matter and nutrients in all supplemented mixtures.

COD removal efficiency ranged from 61.5 to 91.8% for *Chlorella*, and 70.5–93.1% for *Scenedesmus*. Total nitrogen removal was very high, reaching 93.4–99.9% for CH and 98.3–99.9% for SC. Total phosphorus removal was also efficient, varying from 84.3 to 99.9% for CH and 84.9–99.6% for SC (Table 3).

Similar findings were reported by Kshirsagar [53], who demonstrated that nutrient uptake during algal growth, whether through assimilation into biomass or accumulation within algal tissues, directly influences nutrient removal efficiency in wastewater. Nitrogen, a key macronutrient in microalgae, represents 1–10% of total biomass and plays a crucial role in regulating lipid content within algal cells [54]. Likewise, phosphorus utilization during phycoremediation contributes to its removal from wastewater, as it is essential for the synthesis of phospholipids, adenosine triphosphate (ATP), and nucleic acids. Microalgae can also sequester excess phosphorus, storing it intracellularly for future incorporation into biomolecules and metabolic pathways.

#### 3.3.2. Reduction in Heavy Metals and Minerals

Table 2 and Table 3 show a marked decrease in metal and mineral concentrations after biotreatment. In DWW, CH removed 21.9–99.7%, and SC 17.6–99.7% of elements such as Ca, Fe, Mg, Mn, K, and Zn. In DWW + vinasse, CH achieved 14.2–67.9% and SC 17.7–99.5% removal for several metals and minerals. In DWW + whey, CH showed high removal (21.3–99.7%) for most elements, while SC achieved 2.6–99.7%, confirming effective metal and mineral reduction by both algal species. Likewise, similar removal efficiencies are observed in DWW + AFW compared to the other treatments.

The microalgae used in this study were highly effective in removing nutrients and minerals from DWW alone and supplemented with vinasse, in the range 14.16–93.38 (CH), and 17.70–99.46 (SC); whey 21.31–99.73 (CH), and 2.58–99.73 (SC) and AFW, where the removal was in the range 3.02–99.96 (CH), and 10.45–99.96 (SC) (Table 3).

The potential of *Chlorella* and *Scenedesmus* species to effectively remove N and P from household wastewater was also highlighted by Göncü et al. [55]. The authors reported that *S*. *quadricauda* removed up to 83.5% ammonia nitrogen (NH_3_–N) and 57.9% orthophosphate (PO_4_^3−^), while *C*. *vulgaris* achieved 76.5% total nitrogen (TN) removal. Two species of *Chlorella* and two species of *Scenedesmus* were cultivated in a photobioreactor using anaerobically digested wastewater by Chen et al. [56]. *C*. *sorokiniana* showed the highest ammonia and phosphate removal (up to 66.2% phosphate). El-Sheekh et al. [13] also obtained significant results: three algal species were highly efficient and had the potential to reduce nitrate, ammonia, phosphate, sulfate, heavy metals (Zn^2+^, Cu^2+^, Mn^2+^, and Fe^2+^), Ca, Mg, Na, and K, after 10 days of algal treatment compared to initial concentrations.

The high nutrient removal obtained in our study (up to 99.96% N, 99.88 P in CH cultured, and up to 99.96 N and 99.56% P in SC cultures) was similar or higher to the values registered by Ortega-Blas et al. [57], using a *Chlorella*–bacteria consortium to treat municipal wastewater, achieving 87.16% N removal, and 94.43% P removal.

Metal ion accumulation in algae may result from surface adsorption and cytoplasmic binding to compounds such as phytochelatins, metallothioneins, and other intracellular ligands [58]. Algal cell walls are semipermeable and have pore sizes ranging from 5 to 20 nm [59]. Moreover, *C. vulgaris* exhibits clear defensive mechanisms, such as the secretion of exopolymeric substances (EPS) [60]. The EPS, mainly composed of polysaccharides and proteins, contains several functional groups that interact with exogenous materials, altering their bioactivity [61,62]. The extensive surface area and strong binding capacity of algal cells further enhance their effectiveness in heavy metal removal from wastewater [63].

Two key patterns can be identified. First, both microalgae species demonstrated the capacity to assimilate and absorb nutrients from the different wastewater mixtures, indicating their metabolic flexibility and potential for nutrient recovery. Second, the improved nutrient profile could be the primary driver leading to an increase in biomass productivity and lipid production in *Chlorella* sp. and *Scenedesmus* sp. These responses were consistent across cultures grown in DWW and supplemented with vinasse, whey, and AFW, highlighting the robustness of the observed effects. Three distinct wastewater types were tested, and in all cases both species exhibited improved biomass and lipid yields, investigated through multiple bioindicators in both microalgae. Similar findings were reported by Sátiro et al. [21], who presented real-scale evidence that inoculation and microalgae–bacteria synergy enhance nitrogen and phosphorus recovery, supporting comparability and reproducibility of our results. In this context, it is essential to emphasize the multiple benefits of this approach: nutrient recovery, wastewater bioremediation, sustainable waste management, and the assimilation of macro and micronutrients—including metals and trace heavy metals—present in wastewater mixtures.

The simultaneous fixation of inorganic carbon through photosynthesis and the decomposition of the organic carbon source results in higher cell growth and a shorter generation time [64]. Such flexibility reflects the mixotrophic condition of *C*. *vulgaris*, which is capable of combining photosynthesis and heterotrophic assimilation, thereby reducing its exclusive dependence on light and promoting growth in environments rich in organic matter. Moreover, the interaction with bacterial communities closes a cycle in which the oxygen produced by microalgae enhances the aerobic degradation of organic matter, while the released nutrients and CO_2_ are reused by the algae [65,66]. The high nutrient-removal rates obtained in this study are consistent with those achieved in pilot-scale systems [21], under similar algal–bacterial conditions. Integrating these effluents into microalgal cultivation systems not only has economic advantages by mitigating disposal pressures but also unlocks opportunities for nutrient recovery and bioresource production. Anagnostopoulou et al. [10] used the same approach in the food-industry wastewater context, supporting the implications of our work at the system-level (reduced cost, reduced CO_2_, integrated valorization, towards circular economy, climate, and water footprint. Moreover, Tang et al. [22] reported findings consistent with those of the present study, showing that nutrient tailoring through wastewater blending enhances both treatment performance and lipid enrichment. Their results further support the scalability of our approach and indicate that it is neither species- nor site-specific.

Furthermore, the wastewater resulting from the bioremediation process of DWW with microalgae, still rich in nutrients, can be used for irrigation in horticulture, after complying with the sanitary regulations in each locality. In this context, after harvesting *C. vulgaris* biomass, Schmuck et al. [25] evaluated the potential reuse of DWW from an Argentine WWTP following bioremediation, by conducting an acute toxicity assay using *L. sativa* (lettuce) seeds exposed to the treated effluent. No significant differences were observed between treatments and the control (*p* < 0.05), indicating the absence of major toxic effects on seed germination. Similarly, Carvalho et al. [67] assessed a Fe-enriched Brazilian DWW–montmorillonite formulation (TechPhos), and reported no phytotoxicity toward *L. sativa*, *G. max* (soy), or *Ozyra sativa* (rice) seeds. Instead, they observed enhanced germination and root growth in lettuce and soy seeds. Furthermore, the TechPhos–sludge composite was proposed as an innovative fertilizer and slow-release nutrient delivery system, offering a dual benefit: reducing nutrient pollution and strengthening their potential for agricultural applications [67]. In another study, Obijianya et al. [68] reported the reuse of nutrient-rich effluents for agricultural purposes, thereby eliminating the need for additional freshwater.

This approach is particularly relevant during dry seasons and contributes to lowering the overall water footprint. Operating with high concentrations of DWW—especially when reused—reduces dependence on freshwater resources and improves both water quality and resource efficiency. Overall, these findings shed light on the advantages of microalgae-based aqueous extracts as eco-friendly bio-stimulants eligible for sustainable agriculture.

### 3.4. Engineering Perspectives on Scalability

From an engineering standpoint, the integration of *Chlorella* sp. MC18 and *Scenedesmus* sp. MJ23-R cultivation with domestic wastewater (DWW) treatment offers a scalable and resource-efficient bioprocessing framework. The demonstrated ability of both strains to grow in 100% DWW, especially when supplemented with vinasse, whey, or agro-food residues, supports modular system design for nutrient recycling and enhanced lipid productivity. The use of bubble column photobioreactors (BC-PBRs) enables low-energy, vertically scalable cultivation platforms that are well-suited for decentralized deployment in urban and agro-industrial settings. The system’s capacity to reduce COD, stabilize pH, and remove macro- and micronutrients while producing a nutrient-rich effluent for agricultural reuse highlights its potential for integrated water–energy–nutrient management. These engineering attributes align with circular economy principles and provide a foundation for replicable, adaptable infrastructure [6]. Future research should focus on strain optimization and reactor design improvements to maximize lipid yields and streamline downstream processing, thereby advancing the viability of microalgae-based biorefineries at commercial scale.

### 3.5. Sustainability Implications

This study presents a sustainable bioprocessing strategy by integrating microalgal cultivation with domestic wastewater (DWW) treatment. The ability of *Chlorella* sp. MC18 and *Scenedesmus* sp. MJ23-R to grow efficiently in 100% DWW, further enhanced by the addition of vinasse, whey, or agro-food waste, demonstrates effective nutrient recycling and biomass valorization. Cultivation in bubble column photobioreactors (BC-PBRs) enabled low-energy, scalable production with significant improvements in biomass and lipid yields. The microalgal treatment process also contributed to environmental remediation by reducing chemical oxygen demand (COD), stabilizing pH, and removing excess nutrients. The resulting nutrient-rich effluent, suitable for agricultural irrigation, supports water reuse and reduces the overall water footprint. These findings align with circular economy principles, offering a closed-loop system that transforms waste into valuable bioproducts [5]. Future research should focus on optimizing microalgal strains for enhanced lipid and fatty acid production to strengthen their role in renewable biofuel development and broaden their applicability in sustainable biotechnology.

## 4. Conclusions

*Chlorella* sp. MC18 and *Scenedesmus* sp. MJ23-R can grow efficiently in 100% domestic wastewater (DWW). Adding vinasse, whey, or agro-food waste to DWW enhances both algal growth and lipid accumulation. Using bubble column photobioreactors enabled scalable, low-energy cultivation with significant gains in biomass and lipid productivity due to improved nutrient profiles. Both strains reduced COD, stabilized pH, and effectively removed macro and micronutrients. The nutrient-rich effluent remaining after bioremediation can be reused for agricultural irrigation, reducing water footprint. Overall, integrating wastewater treatment with microalgal cultivation supports circular economy principles and offers a sustainable approach for environmental remediation and biomass valorization. Future research should prioritize the bioengineering of microalgae for higher lipid and fatty acid yields to strengthen their role as renewable biofuel feedstocks while also supporting a wide range of biotechnological applications.

## Figures and Tables

**Table 1:** Characteristics of domestic wastewater (DWW), vinasse, whey, and agro-food waste (AFW) samples used in this study. Mean ± standard deviation (±SD) based on *n* = 3 analytical replicates.

Characteristics	Parameters	Unit	DWW	Vinasse	Whey	AFW
pH			6.80 ± 0.11	3.47 ± 0.12	5.05 ± 0.08	4.29 ± 0.05
EC		mS cm^−1^	0.45 ± 0.03	26.89 ± 0.07	6.88 ± 0.07	3.06 ± 0.01
True color		UC	21.7 ± 1.4	40,921 ± 428	1103 ± 88	781 ± 57
Total suspended solids	TSS	mg L^−1^	<2.5	<2.5	<2.5	<2.5
Chemical oxygen demand	COD	mg L^−1^	256 ± 13	74,815 ± 1908	69,003 ± 1607	80,482 ± 2739
Total nitrogen	TN	mg L^−1^	14.8 ± 0.9	1376 ± 91	194 ± 16	102 ± 8
Total phosphorus	TP	mg L^−1^	2.5 ± 0.2	120 ± 7	419 ± 25	46.2 ± 3.4
Boron	B	mg L^−1^	0.0202 ± 0.0010	9.7 ± 0.6	1.63 ± 0.11	2.33 ± 0.15
Calcium	Ca	mg L^−1^	40.3 ± 2.1	920 ± 52	356 ± 23	22.5 ± 1.5
Cobalt	Co	mg L^−1^	<0.00004	0.0364 ± 0.0021	<0.00004	<0.00004
Copper	Cu	mg L^−1^	<0.00004	0.49 ± 0.03	0.0052 ± 0.0004	0.13582 ± 0.00873
Iron	Fe	mg L^−1^	0.032 ± 0.002	94 ± 6	0.049 ± 0.003	0.25774 ± 0.01656
Lithium	Li	mg L^−1^	0.00144 ± 0.00008	1.65 ± 0.09	0.10 ± 0.01	0.01853 ± 0.00119
Magnesium	Mg	mg L^−1^	2.52 ± 0.13	225 ± 13	76 ± 5	56.2 ± 3.6
Manganese	Mn	mg L^−1^	0.0434 ± 0.0022	1.54 ± 0.09	<0.00006	0.33554 ± 0.02156
Molybdenum	Mo	mg L^−1^	<0.00004	0.051 ± 0.003	0.0138 ± 0.0009	0.00511 ± 0.00033
Nickel	Ni	mg L^−1^	<0.00004	0.186 ± 0.011	<0.00002	0.01952 ± 0.00125
Potassium	K	mg L^−1^	8.86 ± 0.45	10,520 ± 598	1679 ± 107	1269 ± 82
Silicon	Si	mg L^−1^	6.3 ± 0.3	32 ± 2	5.8 ± 0.4	7.55 ± 0.49
Sodium	Na	mg L^−1^	23.4 ± 1.2	339 ± 19	564 ± 36	28.5 ± 1.8
Zinc	Zn	mg L^−1^	0.00237 ± 0.00012	3.18 ± 0.18	0.0967 ± 0.0062	0.38574 ± 0.02479

**Table 2:** Physicochemical composition of domestic wastewater (DWW, 100:0 *v*/*v*) and DWW mixture formulations with vinasse, whey and agro-food waste (AFW) (90:10 *v*/*v*, respectively), before and after the cultivation of *Chlorella* sp. MC18 (CH) and *Scenedesmus* sp. MJ23-R (SC) using 4.5 L BC-PBRs.

Parameters	Unit	DWW (100:0, *v*/*v*)			DWW + Vinasse (90:10, *v*/*v*)			DWW + Whey (90:10, *v*/*v*)			DWW + AFW (90:10, *v*/*v*)		
		Value	Algae Treatment		Value	Algae Treatment		Value	Algae Treatment		Value	Algae Treatment	
			CH	SC		CH	SC		CH	SC		CH	SC
pH		6.80	9.85	9.74	7.17	8.39	8.57	7.20	8.00	8.46	7.19	7.75	7.98
EC	mS cm^−1^	0.45	0.33	0.32	4.45	4.58	4.84	1.72	1.86	1.87	0.89	0.93	0.95
True color	UC	21.7	26	14	4129	4492	3872	55	60	76	83	85	91
TSS	mg L^−1^	<2.5	9.3	15.2	<2.5	19.7	16.5	<2.5	24.8	21.9	<2.5	10.4	16.1
COD	mg L^−1^	256	61	17.6	7581	1918	1967	7069	582	496	8254	3176	2438
TN	mg L^−1^	14.8	0.183	0.095	154.0	10.2	2.7	34.8	1.2	0.861	25.3	<0.01	<0.01
TP	mg L^−1^	2.5	<0.003	0.011	14.6	2.3	2.2	42.0	2.6	4.3	7.80	0.43	0.062
B	mg L^−1^	0.0202	0.095	0.079	1.13	0.97	0.93	0.194	0.240	0.189	0.265	0.257	0.280
Ca	mg L^−1^	40.3	6.51	6.75	140.62	57.4	55.4	74.5	46.2	47.0	38.6	26.9	30.5
Co	mg L^−1^	<0.00004	<0.00004	<0.00004	0.00415	0.00317	0.00291	<0.00004	<0.00004	<0.00004	<0.00004	0.000043	0.000045
Cu	mg L^−1^	<0.00004	<0.00004	<0.00004	0.0560	0.0272	0.0314	0.00060	<0.00004	0.00328	0.0145	0.0091	0.0074
Fe	mg L^−1^	0.032	<0.00009	<0.00009	10.8	7.7	7.3	0.03390	<0.00009	<0.00009	0.057	0.048	0.035
Li	mg L^−1^	0.00144	0.00334	<0.00005	0.190	0.138	0.130	0.0120	0.0090	0.0065	0.00328	0.0039	0.0034
Mg	mg L^−1^	2.52	0.87	1.94	27.8	20.0	19.4	10.5	6.1	6.1	8.30	5.60	5.04
Mn	mg L^−1^	0.0434	0.00862	<0.00006	0.2170	0.0697	0.0988	0.0386	0.00493	0.00687	0.0741	0.0278	0.0352
Mo	mg L^−1^	<0.00004	0.0047	0.0045	0.0060	0.0074	0.0078	0.0015	0.0043	0.0054	0.00062	0.0027	0.0025
Ni	mg L^−1^	<0.00002	<0.00002	<0.00002	0.0240	0.0105	0.0112	<0.00002	<0.00002	<0.00002	0.0019	0.00093	0.00106
K	mg L^−1^	8.86	6.92	7.30	1205	963	899	190.0	134.0	144.0	141.6	121.4	126.8
Si	mg L^−1^	6.3	7.2	5.3	9.30	4.3	<0.05	6.1	4.8	5.8	6.5	7.2	7.4
Na	mg L^−1^	23.4	41.0	35.1	59.0	319.0	296.0	82.3	225.0	230.0	24.2	58.6	63.1
Zn	mg L^−1^	0.00237	0.00138	<0.00004	0.3650	0.1289	0.1302	0.01260	0.00323	0.01510	0.0440	0.0319	0.0167

**Table 3:** Macronutrient and micronutrient *i* removal efficiency (*REi*, %) after 8 days of cultivation of *Chlorella* sp. MC18 (CH) and *Scenedesmus* sp. MJ23-R (SC) in 4.5 L BC-PBRs in domestic wastewater (DWW, 100:0 *v*/*v*) and in different formulations of DWW mixtures with vinasse, whey, and agro-food waste (AFW) (90:10 *v*/*v*, respectively).

Parameters	Unit	DWW		DWW + Vinasse		DWW + Whey		DWW + AFW	
		100:0 (*v*/*v*)		90:10 (*v*/*v*)		90:10 (*v*/*v*)		90:10 (*v*/*v*)	
		CH	SC	CH	SC	CH	SC	CH	SC
*RE_COD_*	%	76.17	93.13	74.70	74.05	91.77	92.98	61.52	70.46
*RE_TN_*	%	98.76	99.36	93.38	98.25	96.55	97.53	99.96	99.96
*RE_TP_*	%	99.88	99.56	84.25	84.93	93.81	89.76	94.49	99.21
*RE_B_*	%	–	–	14.16	17.70	–	2.58	3.02	–
*RE_Ca_*	%	83.85	83.25	59.18	60.60	37.99	36.91	30.31	20.98
*RE_Co_*	%	–	–	23.61	29.88	–	–	–	–
*RE_Cu_*	%	–	–	51.43	43.93	93.33	–	37.24	48.97
*RE_Fe_*	%	99.72	99.72	28.70	32.41	99.73	99.73	15.79	38.60
*RE_Li_*	%	–	96.53	27.37	31.58	25.00	45.83	–	–
*RE_Mg_*	%	65.48	23.02	28.06	30.22	41.90	40.00	32.53	39.28
*RE_Mn_*	%	80.14	99.86	67.88	54.47	87.23	82.20	62.48	52.50
*RE_Mo_*	%	–	–	–	–	–	–	–	–
*RE_Ni_*	%	–	–	56.25	53.33	–	–	51.05	44.21
*RE_K_*	%	21.90	17.61	20.08	25.39	29.47	24.21	14.27	10.45
*RE_Si_*	%	–	15.87	53.76	99.46	21.31	4.92	–	–
*RE_Na_*	%	–	–	–	–	–	–	–	–
*RE_Zn_*	%	41.77	98.31	64.68	64.33	74.37	–	27.50	62.05

## Data Availability

Data will be available upon reasonable request and permission of the first and corresponding author.
